# Supplementary material for: Non-culprit left main coronary artery disease in acute myocardial infarction complicated by cardiogenic shock
Source: PLoS One. 2023 Mar 30;18(3):e0276711. doi: 10.1371/journal.pone.0276711 (PMC10062631; doi:10.1371/journal.pone.0276711)
Supplement: S1 File — (DOC) [file pone.0276711.s001.doc]

**SUPPLEMENTAL MATERIAL**

**1) Supplemental Tables**

**1) Supplemental Tables**

| **Supplemental Table 1. 30-day mortality and Mid-term Clinical Outcomes** | | | | | | |
| --- | --- | --- | --- | --- | --- | --- |
|  | **LMCAD non-culprit** | **no LMCAD** | **unadjusted HR** | ***p* value** | **²adjusted HR** | ***p* value** |
|  | **n = 43** | **n = 386** | **95% CI** |  | **95% CI** |  |
| ***30 days all-cause death*** | 16 (37.2) | 114 (29.5) | 1.28 (0.76 - 2.17) | 0.349 | 1.17 (0.68 - 2.01) | 0.580 |
| **6-month follow-up clinical outcomes** | | | | | | |
| **All-cause death** | 17 (39.5) | 125 (32.4) | 1.25 (0.76 - 2.08) | 0.381 | 1.15 (0.68 - 1.94) | 0.605 |
| **Cardiac death** | 13 (30.2) | 103 (26.7) | 1.15 (0.65 - 2.05) | 0.633 | 1.08 (0.59 - 1.96) | 0.809 |
| **Myocardial infarction** | 1 (2.3) | 7 (1.8) | 1.35 (0.17 - 10.96) | 0.780 | 1.03 (0.12 - 9.11) | 0.979 |
| **Repeat revascularization** | 0 (0) | 9 (2.3) | ∽ | ∽ | ∽ | ∽ |
| **¹MACE** | 14 (32.6) | 118 (30.6) | 1.10 (0.63 - 1.92) | 0.728 | 1.00 (0.56 - 1.77) | 0.993 |
| **Re-hospitalization due to HF** | 1 (2.3) | 16 (4.1) | 0.63 (0.08 - 4.74) | 0.653 | 0.82 (0.10 - 6.63) | 0.849 |
| Data are n (%), unless otherwise stated. | | | | | | |
| ¹MACE was defined as a composite of cardiac death, myocardial infarction, and repeat revascularization. | | | | | | |
| ²Adjusted covariates include age ≥ 65 years, sex, diabetes mellitus, current smoking, number of vessel disease, number of used stent, and thrombus aspiration. | | | | | | |
| CI = confidence interval; HF = heart failure; HR = hazard ratio; LMCAD = left main coronary artery disease; MACE = major adverse cardiac event. | | | | | | |

| **Supplemental Table 2. Baseline clinical characteristics and In-hospital management of No event group and Censored data group** | | | |
| --- | --- | --- | --- |
|  | **No event (n=123)** | **Censored data**  **(n=138)** | ***p* value** |
| ***LMCAD*** |  |  | 0.469 |
| no LMCAD | 109 (88.6) | 126 (91.3) |  |
| LMCAD non-culprit | 14 (11.4) | 12 (8.7) |  |
| Age, *years* | 66.3 ± 9.9 | 66.5 ± 1.1 | 0.928 |
| Male | 90 (73.2) | 101 (73.2) | 0.997 |
| Body mass index, *Kg/m²* | 23.6 ± 3.4 | 23.8 ± 3.2 | 0.576 |
| ***Cardiovascular risk factor*** |  |  |  |
| Hypertension | 78 (63.4) | 75 (54.4) | 0.138 |
| Diabetes mellitus | 47 (38.2) | 56 (40.4) | 0.696 |
| Dyslipidemia | 42 (34.2) | 52 (37.7) | 0.553 |
| Chronic renal failure | 7 (5.7) | 10 (7.3) | 0.611 |
| Current smoker | 42 (34.2) | 56 (40.6) | 0.284 |
| Previous PCI | 13 (10.6) | 13 (9.4) | 0.757 |
| Previous myocardial infarction | 13 (10.6) | 11 (8.0) | 0.468 |
| Peripheral artery disease | 3 (2.4) | 6 (4.4) | 0.399 |
| Previous history of stroke | 1 (8.9) | 10 (7.3) | 0.615 |
| ***Clinical presentation*** |  |  |  |
| *Type of acute MI* |  |  | 0.914 |
| non-STEMI | 42 (34.2) | 48 (34.8) |  |
| STEMI | 81(65.9) | 90 (65.2) |  |
| Left ventricular EF, *%* | 42.4 ± 1.3 | 38.1 ± 15.0 | 0.025 |
| Left ventricular EF ≤30% | 36 (29.3) | 51 (37.0) | 0.188 |
| Systolic blood pressure, *mmHg* | 77.2 ± 21.3 | 81.4 ± 27.6 | 0.170 |
| Diastolic blood pressure, *mmHg* | 49.1 ± 16.0 | 51.9 ± 19.4 | 0.219 |
| Heart rate, *beat/min* | 75.1 ± 31.0 | 84.1 ± 31.5 | 0.024 |
| ***Laboratory findings*** |  |  |  |
| Hemoglobin, *g/dL* | 13.4 ± 2.1 | 13.2 ± 2.3 | 0.450 |
| Creatinine*, mg/dL* | 1.3 ± 0.9 | 1.4 ± 1.2 | 0.292 |
| Glucose, *mg/dL* | 221.1 ± 113.4 | 219.8 ± 102.8 | 0.927 |
| Lactic acid, *mmol/L* | 4.6 ± 3.5 | 5.6 ± 4.4 | 0.129 |
| Peak CK-MB, ng/mL | 131.0 (39.0 - 274.0) | 204.3 (62.6 - 300.0) | 0.359 |
| Peak Troponin I, *ng/mL* | 10.2 (0.9 - 50.0) | 17.6 (2.6 - 83.6) | 0.508 |
| ***Emergent in-hospital management*** |  |  |  |
| Undergoing CPR | 13 (10.6) | 18 (13.0) | 0.537 |
| Vasoactive inotropic score | 20.0 (8.0 - 50.0) | 51.8 (20.0 - 202.1) | 0.774 |
| Mechanical ventilation | 53 (43.1) | 60 (43.5) | 0.950 |
| Requiring renal-replacement therapy | 9 (7.3) | 16 (11.6) | 0.241 |
| Requiring ECMO support | 21 (17.1) | 32 (23.2) | 0.220 |
| Data are n (%), mean ± standard deviation, or median (interquartile range). | | | |
| CK-MB = creatine kinase myocardial band; CPR = cardiopulmonary resuscitation; ECMO = extracorporeal membrane oxygenation; EF = ejection fraction; LMCAD = left main coronary artery disease; MI = myocardial infarction; PCI = percutaneous coronary intervention; STEMI = ST-segment elevation myocardial infarction. | | | |

| **Supplemental Table 3. Angiographic and Procedural characteristics of No event group and Censored data group** | | | |
| --- | --- | --- | --- |
|  | **No event (n=123)** | **Censored data**  **(n=138)** | ***p* value** |
| ***Angiographic findings*** |  |  |  |
| *Culprit lesion location* |  |  | 0.081 |
| LAD | 51 (41.5) | 68 (49.3) |  |
| LCX | 13 (10.6) | 22 (15.9) |  |
| RCA | 59 (48.0) | 48 (34.8) |  |
| *Culprit lesion TIMI flow grade, pre-PCI* | |  | 0.060 |
| 0 | 71 (57.7) | 70 (50.7) |  |
| 1 | 13 (10.6) | 11 (8.0) |  |
| 2 | 24 (19.5) | 22 (15.9) |  |
| 3 | 15 (12.2) | 35 (25.4) |  |
| *Culprit lesion TIMI flow grade, post-PCI* | |  | 0.800 |
| *0* | 1 (0.8) | 1 (0.7) |  |
| 1 | 1 (0.8) | 3 (2.2) |  |
| 2 | 14 (11.4) | 18 (13.0) |  |
| 3 | 107 (87.0) | 116 (84.1) |  |
| *Number of diseased coronary vessel* |  |  | 0.711 |
| 2-vessel disease | 73 (59.4) | 85 (61.6) |  |
| 3-vessel disease | 50 (40.7) | 53 (38.4) |  |
| Number of stenotic lesions | 2.7 ± 1.0 | 2.6 ± 0.9 | 0.307 |
| SYNTAX score, pre-PCI | 23.0 ± 10.6 | 22.1 ± 9.4 | 0.474 |
| SYNTAX score, post-PCI | 7.1 ± 8.1 | 7.1 ± 6.4 | 0.975 |
| ***Procedural characteristics*** |  |  |  |
| *Access site* |  |  | 0.362 |
| Transradial approach | 96 (78.1) | 101 (73.2) |  |
| Transfemoral approach | 27 (22.0) | 37 (26.8) |  |
| Number of used stent | 1.5 ± 0.9 | 1.4 ± 0.8 | 0.208 |
| Contrast volume, mL | 180.5 ± 65.2 | 184.0 ± 64.1 | 0.840 |
| Thrombus aspiration | 41 (33.3) | 42 (30.4) | 0.616 |
| Glycoprotein IIb/IIIa inhibitor | 20 (16.3) | 35 (25.4) | 0.072 |
| Performed staged PCI | 17 (13.8) | 13 (9.4) | 0.266 |
| Data are n (%), or mean ± standard deviation. | | | |
| LAD = left anterior descending artery; LCX = left circumflex artery; LMCAD = left main coronary artery disease; PCI = percutaneous coronary intervention; RCA = right coronary artery; SYNTAX = Synergy between PCI with Taxus and Cardiac Surgery; TIMI = thrombolysis in myocardial infarction. | | | |
