# Supplementary material for: Non-culprit left main coronary artery disease in acute myocardial infarction complicated by cardiogenic shock
Source: PLoS One. 2023 Mar 30;18(3):e0276711. doi: 10.1371/journal.pone.0276711 (PMC10062631; doi:10.1371/journal.pone.0276711)
Supplement: S1 Protocol — (PDF) [file pone.0276711.s002.pdf]

STUDY PROTOCOL

Study title: [**SM**art **A**ngioplasty **R**esearch **T**eam: A Multi-center, open, **RE**trospective and prospective observational **S**tudy to investigate **C**linical o**U**tcomes and **E**fficacy of left ventricular assist device for Korean patients with cardiogenic shock : **RESCUE**]

Protocol No.: Ver 2.6

Written date: August 10, 2017

INDEX

Summary of clinical trial....3
1. Name and phase of clinical trial....6
2. Name and address of participating centers (Attachment 1)....6
3. Principal investigator, sub-investigator, and co-investigator (Attachment 2)....6
4. Clinical trial pharmacist of investigational product....6
5. Name and address of clinical trial sponsor....6
6. Purpose and background of clinical trial....6
7. Medical device information....8
8. Target disease....8
9. Inclusion criteria, exclusion criteria, and targeted sample size and their basis....8
10. Duration of clinical trial....10
11. Clinical trial method....10
12. Observation items/clinical examination items and observation/testing methods....11
13. Predicted side effects and precautions for use....12
14. Stopping/dropping criteria....12
15. Evaluation criteria for effectiveness, and evaluation and interpretation methods (statistical analysis)....13
16. Evaluation criteria for safety including side effects, and evaluation and reporting methods....14
17. Indemnity procedures for any harm....14
18. Criteria for the medical treatment of subjects after the clinical trial....14
19. Measures for ensuring the safety of subjects....14
20. Other necessary matters for conducting the clinical trial safely and scientifically....15
REFERENCE....16
Attachment 1. Name and address of participating centers....17
Attachment 2. Principal investigator, sub-investigator, and co-investigator....17

Summary of clinical trial

Name	A Multi-center, open, REtrospective and prospective observational Study to investigate Clinical oUtcomes and Efficacy of left ventricular assist device for Korean patients with cardiogenic shock : RESCUE
Purpose	The purpose of this study is to investigate the treatment status and clinical outcomes of cardiogenic shock in Koreans, as well as identify factors that can improve prognosis through retrospective and prospective registration of patients with cardiogenic shock from multiple institutions in Korea. In addition, we aim to determine the current usage and treatment effects of IABP and ECMO for cardiogenic shock.
Institution	Samsung medical center
Principal investigator	Hyeon-Cheol Gwon, Professor, Samsung Medical Center<br>Jeong Hoon Yang, Professor, Samsung Medical Center
Duration	IRB approval date ~ December 31, 2019 (registration period: IRB approval date ~ December 31, 2018).<br>Data collection period: January 1, 2014 ~ December 31, 2019 (6 years).
Design	Retrospective and prospective, open, multi-center, and observational registry study
Estimated enrollemnt	1000 participants (competitive registration after registering 1,000 participants)<br>· The group with left ventricular assist device insertion: more than 400 participants<br>· The group without left ventricular assist device insertion: more than 600 participants
Inclusion & exclusion criteria	[**Inclusion criteria**]<br>1. 19 years old or older<br>2-1). Systolic blood pressure is less than 90mmHg for more than 30 minutes despite the fluid therapy, or Use of pressure boosting agents is necessary.<br>2-2). Peripheral hypopnea (cold skin, urine less than 30 cc per hour, impaired consciousness, lactate ≥2.0 mmol / l) or a person with pulmonary edema.<br>3. Causes of cardiogenic shock include acute myocardial infarction (including in coronary intervention), cardiomyopathy, myocarditis, pulmonary embolism, refractory ventricular tachycardia, shock during coronary intervention.<br>4. In-hospital cardiac arrest as a result of the cause stated in 'inclusion criteria 3'.<br>5. Those voluntarily consenting to the medical records and the data necessary for the study during the entire study period.<br><br>[**Exclusion criteria**]<br>1. Other causes except for cardiogenic shock (low blood pressure, septic, obstructive shock)

	2. Shock with cardiac arrest outside the hospital<br>3. People with allergic reactions to anticoagulants such as heparin<br>4. Those who refused active treatment
Detailed description	1) The selection / exclusion criteria for persons with cardiogenic shock should be verified by medical records from January 1, 2014 before the approval date of each institution's clinical trial screening committee.<br>2) If a cardiogenic shock to the selection criteria is found in the emergency room, general ward, or intensive care unit of the participating institutions, enroll in this study and fill in the information according to the e-CRF.<br>3) After 1, 6 and 12 months, visit the hospital for examination and procedures. The person in charge of the examination or the delegate of the examiner may follow the person by telephone or an outpatient visit.
Outcome measures	**Primary Endpoint**<br>In-hospital death<br><br>**Secondary Endpoints**<br>1. Death in 28 days<br>2. Death during follow-up<br>3. Death, myocardial infarction, stroke, re-admission due to heart failure during follow-up<br>4. Number of days of intensive care unit<br>5. Hospitalization days<br>6. Lactate 24 hour clearance<br>7. Successful removal of left ventricular assist device (IABP, ECMO)

Clinical trial flow chart

	Visit 1 (admission)	Follow-up*		
		Visit 2 (1M)	Visit 3 (6M)	Visit 4 (12M)
		4 Week ±1wk	24 Week ±4wks	48 Week ±4wks
Obtaining consent	X[1]			
Inclusion/exclusion criteria	X			
Height and weight/vital signs	X			
Demographic information and risk factors/medical history	X[2]			
Medication history	X			
Investigation of cardiovascular event	X	X	X	X
Coronary angiography	X			
Lt. ventricular assist device insertion	X			
Complications related to the procedure[3]	X			
Echocardiography	X			
General blood chemistry test	X[4]		X[4]	
Lactate, ScVO2	X[5]			
CPK, CK-MB, Troponin, NT-ProBNP	X		X	

1. There must be a signature from the patient or their legal guardian on the consent form. As this study is a retrospective and prospective registry study, data can be collected from patients who meet the selection criteria for either undergoing coronary angiography, insertion or non-insertion of a left ventricular assist device, after obtaining consent for access to medical records.
2. Age, sex, risk factors, medical history, clinical diagnosis, etc.
3. Investigate whether complications such as limb ischemia, stroke, sepsis, major bleeding events, vascular injury during insertion, and failure of left ventricular assist device insertion occurred.
4. CBC, Creatinine, Fasting glucose level, AST, ALT, T-bilirubin, LDH, Sodium
5. Lactate and ScVO2 are measured every 6 hours from the onset of shock, and then once a day until shock recovery.

* Telephone (landline) interviews are also allowed for information gathering without the subjects having to visit the hospital.

1. Name and phase of clinical trial

SMart **A**ngioplasty **R**esearch **T**eam: A Multi-center, open, **RE**trospective and prospective observational **S**tudy to investigate **C**linical o**U**tcomes and **E**fficacy of left ventricular assist device for Korean patients with cardiogenic shock : **RESCUE**

2. Name and address of participating centers (Attachment 1)

3. Principal investigator, sub-investigator, and co-investigator (Attachment 2)

4. Clinical trial pharmacist of investigational product: Not applicable

5. Name and address of clinical trial sponsor

Hyeon-Cheol Gwon, Division of Cardiology, Heart Vascular Stroke Institute, Samsung Medical Center, Sungkyunkwan University School of Medicine, 81 Irwon-ro

Jeong Hoon Yang, Division of Cardiology, Department of Critical Care Medicine and Medicine, Sungkyunkwan University School of Medicine, 81 Irwon-ro

6. Purpose and background of clinical trial

6.1. Purpose

The purpose of this study is to investigate the treatment status and clinical outcomes of cardiogenic shock in Koreans, as well as identify factors that can improve prognosis through retrospective and prospective registration of patients with cardiogenic shock from multiple institutions in Korea. In addition, we aim to determine the current usage and treatment effects of IABP and ECMO for cardiogenic shock.

6.2. Background

Cardiogenic shock usually occurs as a fatal complication of acute myocardial infarction, cardiomyopathy, myocarditis, and rarely, sepsis. In particular, shock occurs in 5-8% of cases of ST-segment elevation myocardial infarction, and despite the development of various treatment techniques such as coronary intervention and coronary artery bypass grafting, a very high mortality rate of about 40% is still reported [1]. Shock is a term that encompasses all clinical situations in which cellular respiration is not properly carried out due to inadequate oxygen supply to tissues. It was first described by a French surgeon in 1737, and much progress has been made in the treatment and research of shock through the two World Wars and the Korean and Vietnam Wars. Types of shock include hypovolemic shock, represented by excessive bleeding due to trauma or surgery, septic shock caused by decreased peripheral vascular resistance due to infection, obstructive shock caused by circulatory disorders such as pulmonary embolism and cardiac tamponade, and cardiogenic shock caused by decreased cardiac output due to loss of cardiac pump function. The prominent characteristic of cardiogenic shock is that while the early mortality rate is the highest, once the patient recovers from shock, they can maintain a good quality of life with a better prognosis compared to other shocks. Currently, most of the studies related to cardiogenic shock published in Korea have selected patients through the arbitrary criterion of Killip class IV in acute myocardial infarction registry, so it is difficult to accurately determine the frequency and treatment status, mortality rate, etc. of cardiogenic shock caused by causes other than myocardial infarction. Since the adequacy of appropriate early treatment within 24 hours after the occurrence of cardiogenic shock determines the prognosis, it is essential to evaluate the treatment status and appropriateness of cardiogenic shock in Korea through research at the present time. In addition, the initial treatment of cardiogenic shock is primarily fluid therapy, mechanical ventilation, and vasopressor therapy, but if these are ineffective, left ventricular assist devices such as intra-aortic balloon pumps (IABP) and extracorporeal membrane oxygenation (ECMO) can be used. However, there are still no large-scale systematic studies in Korea to determine their effects.

Recently, in a study conducted by Thiele et al., the superiority of IABP treatment was not demonstrated compared to standard treatment group in patients with cardiogenic shock accompanied by acute myocardial infarction for whom early coronary intervention was scheduled [2]. As a result, research on the effectiveness and safety of mechanical circulatory support devices that can more effectively assist left ventricular function in cardiogenic shock is being actively pursued worldwide [3-6]. In particular, the expected benefits of ECMO, a left ventricular assist device that can be operated anywhere in emergency situations, are significant, and interest in ECMO procedures is rapidly increasing in Korea, with the number of procedures increasing rapidly. Therefore, it is urgent to evaluate the appropriateness and effectiveness of ECMO and conduct research on its effects.

7. Medical device information

- ECMO: The Capiox Emergency Bypass System (Terumo, Tokyo, Japan) or Prolonged Life Support System (Maquet Cardiopulmonary AG, Hirrlingen, Germany)
- IABP (Datascope or Arrow)

8. Target disease

Cardiogenic shock

9. Inclusion criteria, exclusion criteria, and targeted sample size and their basis

9.1. Inclusion criteria

1. 19 years old or older

2-1). Systolic blood pressure is less than 90mmHg for more than 30 minutes despite the fluid therapy, or Use of pressure boosting agents is necessary.

2-2). Peripheral hypopnea (cold skin, urine less than 30 cc per hour, impaired consciousness, lactate ≥2.0 mmol / l) or a person with pulmonary edema.

3. Causes of cardiogenic shock include acute myocardial infarction (including in coronary intervention), cardiomyopathy, myocarditis, pulmonary embolism, refractory ventricular tachycardia, shock during coronary intervention.

4. In-hospital cardiac arrest as a result of the cause stated in 'inclusion criteria 3'.

5. Those voluntarily consenting to the medical records and the data necessary for the study during the entire study period.

9.2. Exclusion criteria

1. Other causes except for cardiogenic shock (low blood pressure, septic, obstructive shock)
2. Shock with cardiac arrest outside the hospital
3. People with allergic reactions to anticoagulants such as heparin
4. Those who refused active treatment

9.3. Targeted sample size and their basis

(1) Estimated enrollment: 1000 participants (competitive registration after registering 1,000 participants)

- The group with left ventricular assist device insertion: more than 400 participants
- The group without left ventricular assist device insertion: more than 600 participants

(2) Background

Samsung Medical Center, Korea University Anam Hospital, Sejong General Hospital, Bucheon, Samsung Changwon Hospital, Ilsan Paik Hospital, Dankook University Medical Center, Konkuk University Medical Center, Chung-Ang University Hospital, Inha University Hospital, and Chungbuk National University Hospital are expected to participate, and considering the fact that 1,800 patients with acute myocardial infarction are being treated annually in 10 hospitals, about 5.5% of myocardial infarction patients progress to shock, which means that about 100 patients can be registered each year, and it is estimated that more than 100 patients can be registered due to other causes such as unstable angina, shock during procedures, cardiomyopathy, and myocarditis. Considering the five-year study period, it is estimated that more than 1,000 patients can be registered.

As for the group with left ventricular assist devices (LVADs) inserted, most of them are critically ill patients, so it should be noted that a comparison with the group without LVADs inserted cannot be made on an equal footing. However, other than that, sample size/power calculations for binomial proportions were applied using Fleiss, Tytun, and Ury (1980). With the above number of patients, it has about 88% statistical power to detect an odds ratio of 1.56 (inserted group = 0.3, non-inserted group = 0.4). However, if an expected dropout rate of about 10% is taken into account, the power is reduced to 84%.

In addition, considering that the group with LVADs inserted is significantly superior to the group without LVADs, a one-sided test is usually performed, and when adjusted, the power exceeds 90%. Therefore, it is expected that this clinical trial plan will be statistically and clinically successful when executed.

Referene:

Fleiss, JL, Tytun A, Ury SHK. (1980). A Simple approximation for calculating sample sizes for comparing independent proportions. Biometrics, 36. 343-346.

*The above power calculation was generated using nQuery Advisor 4.0.

10. Duration of clinical trial

IRB approval date ~ December 31, 2019 (registration period: IRB approval date ~ December 31, 2018).

Data collection period: January 1, 2014 ~ December 31, 2019 (6 years).

11. Clinical trial method

11.1. Overview

Shock patients with cardiogenic shock caused by acute myocardial infarction, myocardial disease, myocarditis, etc. will be registered both retrospectively and prospectively for two and a half years before and after IRB approval. Patients undergoing coronary intervention and treatment are performed according to the standard method recommended by the Korean Society of Cardiology. If the patient satisfies the above conditions and meets other inclusion and exclusion criteria, we obtain the patient's consent and enroll them in the study. On the day of shock occurrence, we perform physical measurements, blood tests, investigate the medication being taken, and record the most severe signs of vitality on that day. The maximum dose of medication used for shock treatment, the insertion of a left ventricular assist device, and indicators of shock deterioration and improvement are followed up. We conduct tests scheduled for the day of shock occurrence and one month, six months, and twelve months afterward to confirm whether a death event occurred. If the subject is unable to visit the hospital due to unavoidable circumstances, the trial responsible person or the person delegated by the trial responsible person may follow-up and check the subject's survival status and death event by phone.

11.2. Insertion of left ventricular assist devices

The insertion of a left ventricular assist device is typically performed according to the recommendations of the American Heart Association and the Korean Society of Cardiology when drugs are not effective. However, in exceptional cases such as myocardial infarction with left ventricular wall motion abnormalities that are expected to result in cardiac arrest, the decision to insert IABP and ECMO more aggressively is left to the discretion of the operator. After the insertion of a left ventricular assist device, anticoagulant therapy should be administered to prevent thromboembolism unless it is contraindicated, and in order to prevent limb ischemia, the insertion of a catheter for limb perfusion is recommended if there is no palpable doppler signal or a change in limb color is detected. If pulmonary edema and pulmonary hemorrhage occur due to left ventricular dilatation after ECMO insertion, percutaneous or surgical decompression is recommended.

11.3. Follow-up schedule

Clinical follow-up: date of shock occurance, 1, 6, 12M

12. Observation items/clinical examination items and observation/testing methods

12.1. Visit 1 (day of shock occurrence)

① Written informed consent for patients enrolled

According to the scope of data collection, it is divided into prospective data collection subjects and retrospective data collection subjects.

- Prospective data collection subjects: The day of shock occurrence is after the IRB approval date
- Retrospective data collection subjects: The day of shock occurrence is before the IRB approval date

For prospective data collection subjects, before entering the clinical trial, the purpose and content of the trial should be explained in detail to the subject or legal guardian (in the case of a subject who cannot provide consent for the trial), and the written consent form should accurately record the signature dates of the subject and investigator. The original signed consent form is kept by the subject, and a copy is provided to the subject. If the subject is unable to consent on the day of the shock occurrence, the consent form is obtained from the representative of the accompanying subject. In this case, the principal investigator and sub-investigator must inform the subject or the subject's representative about the clinical trial as soon as possible and obtain consent to continue participating in the trial. If the subject recovers to a condition where consent can be given, the consent form is obtained from the subject.

For retrospective data collection subjects, only data corresponding to the study protocol are collected until IRB approval is obtained without written consent. However, among shock occurrence subjects before IRB approval, those who have obtained written consent are eligible for both prospective and retrospective data collection.

② Inclusion/exclusion criteria

Subjects should be reviewed by the investigator or their delegate to ensure that the subject is suitable for the study.

③ Demographic information and risk factor/medical history surveys

Demographic information surveys of the clinical trial subjects are conducted. Recorded items include subject sex, age, risk factors, medical history related to cardiovascular disease, clinical diagnosis, and other past medical history.

④ Physical measurements and vital signs

Height and weight are measured. Vital signs including blood pressure, and heart rate are measured before any other planned tests are conducted.

⑤ Clinical laboratory test

CBC	Hemoglobin, WBC, Platelets
Chemistry profile	Creatinine, Fasting glucose level, AST,ALT, T-bilirubin, LDH, Sodium
Perfusion	Lactate, ScVO2
Cardiac enzymes	CPK, CK-MB, Troponin I, NT-proBNP

⑥ Medication history

The medication history is investigated through interviews and past medical records.

⑦ Survival and death event investigation

⑧ Echocardiography

⑨ Coronary angiography for ischemic heart disease

⑩ Insertion of left ventricular assist device and investigation of complications

12.2. Follow-up

Visits are conducted at 1 month, 6 months, and 12 months after the shock event for the following tests and procedures. Information gathering through phone (landline contact) is also allowed without the subject's visit.

① Survival and death event investigation

② laboratory tests (conducted at 6 months).

CBC	Hemoglobin, WBC, Platelets
Chemistry profile	Creatinine, Fasting glucose level, AST,ALT, T-bilirubin, LDH, Sodium
Cardiac enzymes	CPK, CK-MB, Troponin I, NT-proBNP

13. Predicted side effects and precautions for use

In cases of ischemic heart disease, standard percutaneous coronary intervention is performed, and the use of left ventricular assist devices to restore signs of vitality in the event of shock is necessary, so there are no increased side effects resulting from this study. Possible complications during the procedure include systemic embolism, including stroke, coronary artery dissection, perforation, rupture, side branch occlusion, stent migration and damage, and acute thrombosis-induced myocardial infarction and ventricular arrhythmia, but they will not increase due to this study. Stent thrombosis can increase due to early discontinuation of antiplatelet agents such as aspirin and P2Y12 inhibitors taken after the procedure.

14. Stopping/dropping criteria

① When the subject or legal guardian requests to withdraw from the clinical trial
② When the subject refuses to comply with the investigator's instructions or fails to comply with the provisions stated in the consent form
③ When the subject's absence makes continuous observation impossible
④ When the subject dies for reasons unrelated to the clinical trial
⑤ Other cases where the clinical trial coordinator determines that there are problems with the conduct of the clinical trial.

Method of handling

① If a subject drops out in the middle of the clinical trial, the reason for dropping out and the data related to the clinical trial up to the point of dropping out shall be recorded and stored.
② Unless there is a valid reason or justification, those who drop out in the middle shall be included in the safety and efficacy evaluation statistics processing.

15. Evaluation criteria for effectiveness, and evaluation and interpretation methods (statistical analysis)

15.1. Primary Endpoint

In-hospital death

15.2. Secondary Endpoints

1) Death in 28 days
2) Death during follow-up
3) Death, myocardial infarction, stroke, re-admission due to heart failure during follow-up
4) Number of days of intensive care unit
5) Hospitalization days
6) Lactate 24 hour clearance
7) Successful removal of left ventricular assist device (IABP, ECMO)

15.3. Statistical analysis

We aim to identify predictors of in-hospital death in patients who experience shock using binary logistic regression analysis. This type of analysis requires 10-15 events per independent variable, and with an expected mortality rate of about 40%, or approximately 400 deaths, we have sufficient events to study various risk factors. The goodness-of-fit of the model will be assessed using the C-statistic and Hosmer-Lemeshow test. In addition, we plan to compare the in-hospital death rate between patients with and without left ventricular assist devices using chi-square tests among those who receive a certain level of vasoactive drugs. We also aim to identify predictors of successful removal of the ECMO device and procedure-related complications such as bleeding, thrombosis, and limb ischemia using binary logistic regression analysis in patients who undergo ECMO. Finally, we plan to evaluate overall death over five years using Cox survival

analysis. We will assess the goodness-of-fit of the model by checking Schoenfeld's residuals and identify the hazard of each risk factor.

16. Evaluation criteria for safety including side effects, and evaluation and reporting methods

Not applicable

17. Indemnity procedures for any harm

By participating in this clinical study, we do not provide any separate compensation for the treatment of predictable or unexpected side effects or complications that may occur. There is no financial compensation provided for participating in this clinical study, and the tests conducted are not tests performed specifically for the study, but rather tests that would be received during routine clinical care, whether or not the individual participates in the study. Therefore, any tests or consultation fees related to the clinical trial are the responsibility of the subject.

18. Criteria for the medical treatment of subjects after the clinical trial

After the 12-month study is completed, participants will continue to periodically visit the outpatient clinic according to each treatment provider's follow-up criteria to receive physical examinations and evaluations. They will also receive ongoing education to seek immediate consultation in case of symptoms such as chest pain, heart failure, or arrhythmia. In the event of adverse reactions or the need for hospitalization, the researchers will make every effort to minimize the risk.

19. Measures for ensuring the safety of subjects

19.1. Protection of subject confidentiality

Records that can identify the subject will be kept confidential and will not be publicly disclosed. However, monitors, inspectors, review boards, and government agencies may directly access the subject's medical records or data within the scope permitted by relevant laws or regulations to verify the implementation procedures and reliability of the data of the clinical trial. Even in this case, confidentiality will be maintained as much as possible. The subject's signature on the consent form indicates permission for such direct access, and the subject's identity will be kept confidential when the results of the clinical trial are published.

Personal information such as the subject's name may be collected as a result of participation in this clinical trial, but this information will only be used for the purpose of linking to clinical information obtained

through the clinical trial and will not be directly used or required for the research. Therefore, the collected information will be used until the completion of the preparation of the clinical trial report and will be appropriately managed in accordance with the Personal Information Protection Act.

19.2. Method of data coding

In all documents related to clinical trials such as case records, the subject's name is recorded and distinguished by the subject identification code (usually Case No.), not the subject's name.

20. Other necessary matters for conducting the clinical trial safely and scientifically

20.1. Changes to the clinical trial protocol

According to KGCP, clinical trials are conducted after obtaining approval from the IRB before the start of the clinical trial, and changes/modifications to the clinical trial protocol are applied only after approval from the clinical trial review committee (IRB).

20.2. Informed consent form

Patients who have heard sufficient explanation about the study and have voluntarily written the informed consent form are eligible to participate in the clinical trial. If there are any changes regarding safety during the study, signatures must be obtained from all participants for the proposed modifications. Researchers must inform patients that they have the right to refuse participation freely and may withdraw from the study at any time without any future disadvantage to their treatment. If the patient is deemed to have understood the research participation sufficiently, the patient can sign the informed consent form. The researcher provides the patient with a copy of the signed informed consent form.

Reference

[1] Thiele H, Allam B, Chatellier G, Schuler G, Lafont A. Shock in acute myocardial infarction: the Cape Horn for trials? European Heart Journal 2010;31:1828-35.

[2] Thiele H, Zeymer U, Neumann FJ, et al. Intra-aortic balloon counterpulsation in acute myocardial infarction complicated by cardiogenic shock (IABP-SHOCK II): final 12 month results of a randomised, open-label trial. Lancet 2013;382:1638-45.

[3] Rousse N, Juthier F, Pincon C, et al. ECMO as a bridge to decision: Recovery, VAD, or heart transplantation? International Journal of Cardiology 2015;187:620-7.

[4] Napp LC, Brehm M, Kuhn C, Schafer A, Bauersachs J. Heart against veno-arterial ECMO: Competition visualized. International Journal of Cardiology 2015;187:164-5.

[5] Knezevic I, Poglajen G, Ksela J, et al. ECMO as a Bridge-to-Transplant in Patients With Cardiogenic Shock. Journal of Heart and Lung Transplantation 2015;34:S314-S5.

[6] Buesing K, Legband N, Goede MR, Borden MA, Terry BS. Alternative to Ecmo: Development of a Novel Peritoneal Membrane Oxygen Delivery System. Shock 2015;43:67-8.

Attachment 1. Name and address of participating centers

	Institutes	Location	Principal investigator
1	Samsung Medical Center	81, Irwon-ro, Gangnam-gu, Seoul	Hyeon-Cheol Gwon/ Jeong Hoon Yang
2	Korea University Anam Hospital	73, Goryeodae-ro, Seongbuk-gu, Seoul	Cheol Woong Yu
3	Sejong General Hospital	489, Hohyeon-ro, Bucheon-si	Je Sang Kim
4	Samsung Changwon Hospital	158, Paryong-ro, Masanhoewon-gu, Changwon-si	Woo Jung Chun
5	Inje University Ilsan Paik Hospital	170, Juhwa-ro, Ilsanseo-gu, Goyang-si	Sung Uk Kwon
6	Dankook University Hospital	201, Manghyang-ro, Dongnam-gu, Cheonan-si	Seong-Hoon Lim
7	Konkuk University Hospital	120-1, Neungdong-ro, Gwangjin-gu, Seoul	Hyun-Joong Kim
8	Chung-Ang University Hospital	102, Heukseok-ro, Dongjak-gu, Seoul	Wang Soo Lee
9	Inha University Hospital	27, Inhang-ro, Jung-gu, Incheon	Sang-Don Park
10	Chungbuk National University Hospital	776, 1sunhwan-ro, Seowon-gu, Cheongju-si	Jang-Whan Bae

Attachment 2. Principal investigator, sub-investigator, and co-investigator

Institutes	Role	Name	Title
Samsung Medical Center	Principal investigator	Hyeon-Cheol Gwon	Cardiology Professor
		Jeong Hoon Yang	Critical care medicine/ Cardiology Professor
	co-investigator	Seung-Hyuk Choi	Cardiology Professor
		Jin Ho Choi	Cardiology Professor
		Joo-Yong Hahn	Cardiology Professor
		Young Bin Song	Cardiology Professor
		Joo Myung Lee	Cardiology Professor
		Taek Kyu Park	Cardiology Fellowship
Korea University Anam Hospital	Principal investigator	Cheol Woong Yu	Cardiology Professor
Sejong General Hospital	Principal investigator	Je Sang Kim	Cardiology Professor
Samsung Changwon Hospital	Principal investigator	Woo Jung Chun	Cardiology Professor
Inje University Ilsan Paik Hospital	Principal investigator	Sung Uk Kwon	Cardiology Professor
Dankook University Hospital	Principal investigator	Seong-Hoon Lim	Cardiology Professor

Konkuk University Hospital	Principal investigator	Hyun-Joong Kim	Cardiology Professor
Chung-Ang University Hospital	Principal investigator	Wang Soo Lee	Cardiology Professor
Inha University Hospital	Principal investigator	Sang-Don Park	Cardiology Professor
Chungbuk National University Hospital	Principal investigator	Jang-Whan Bae	Cardiology Professor
